# Proofreading Activity of DNA Polymerase Pol2 Mediates 3′-End Processing during Nonhomologous End Joining in Yeast

**DOI:** 10.1371/journal.pgen.1000060

**Published:** 2008-04-25

**Authors:** Shun-Fu Tseng, Abram Gabriel, Shu-Chun Teng

**Affiliations:** 1 Department of Microbiology, College of Medicine, National Taiwan University, Taipei, Taiwan; 2 Department of Biochemistry and Molecular Biology, Rutgers University, Piscataway, New Jersey, United States of America; 3 Institute of Internal Medicine, National Taiwan University Hospital, Taipei, Taiwan; Brandeis University, United States of America

## Abstract

Genotoxic agents that cause double-strand breaks (DSBs) often generate damage at the break termini. Processing enzymes, including nucleases and polymerases, must remove damaged bases and/or add new bases before completion of repair. Artemis is a nuclease involved in mammalian nonhomologous end joining (NHEJ), but in *Saccharomyces cerevisiae* the nucleases and polymerases involved in NHEJ pathways are poorly understood. Only Pol4 has been shown to fill the gap that may form by imprecise pairing of overhanging 3′ DNA ends. We previously developed a chromosomal DSB assay in yeast to study factors involved in NHEJ. Here, we use this system to examine DNA polymerases required for NHEJ in yeast. We demonstrate that Pol2 is another major DNA polymerase involved in imprecise end joining. Pol1 modulates both imprecise end joining and more complex chromosomal rearrangements, and Pol3 is primarily involved in NHEJ-mediated chromosomal rearrangements. While Pol4 is the major polymerase to fill the gap that may form by imprecise pairing of overhanging 3′ DNA ends, Pol2 is important for the recession of 3′ flaps that can form during imprecise pairing. Indeed, a mutation in the 3′-5′ exonuclease domain of Pol2 dramatically reduces the frequency of end joins formed with initial 3′ flaps. Thus, Pol2 performs a key 3′ end-processing step in NHEJ.

## Author Summary

Chromosomal DSBs caused by replication fork disruption, environmental factors, or endogenous nucleases are common yet potentially dangerous DNA lesions in all organisms. If they are repaired by homologous recombination (HR), the integrity of the genome is usually maintained. However, if the broken ends undergo NHEJ, sequences at the junction may be added, deleted, or substituted, and large segments of chromosomes can be rearranged. Partially overlapping sets of proteins are required for repair by either the HR or NHEJ pathway. Furthermore, different proteins may be used to process broken DNA ends, depending on the particular terminal structures. Since DNA synthesis occurs during HR in yeast and all three essential replicative polymerases are utilized, we asked how different polymerases might be involved in DSB repair by NHEJ. We find that Pol2, and particularly the enzyme's 3′ to 5′ nuclease activity, contributes to the removal of 3′ single strand flaps that can form during the initial joining of broken ends. We find that Pol1 and Pol3 modulate complex chromosomal rearrangements, and we confirm that Pol4 fills the gap that can form by imprecise pairing of overhanging 3′ DNA ends. Our work demonstrates that multiple DNA polymerases play important roles in NHEJ.

## Introduction

DNA DSBs result from disruption of the phosphodiester backbone on both strands of a DNA double helix. They are induced by ionizing radiation and chemicals, including anticancer drugs, or can arise spontaneously during DNA replication [1]–[3]. Furthermore, DSBs occur normally as intermediates in V(D)J recombination, the process that helps to generate the vast range of antigen-binding sites of antibody and T-cell receptor proteins during lymphoid-cell development [4]. DNA DSBs are critical lesions that, if unrepaired or misrepaired, may be lethal for a cell or help in its malignant transformation.

DSBs can be repaired either by homologous recombination (HR) or by NHEJ [1], [3], [5]–[8]. While the former process is generally error-free, the latter process is potentially error-prone. DSB repair by HR requires extensive regions of sequence homology between donor and recipient DNA strands. In NHEJ the DNA ends are joined with little or no base pairing at the junction [9]. These repair mechanisms are evolutionarily conserved, but contribute unequally to overall DSB repair in different organisms. In mammals, DSBs are primarily repaired by NHEJ, while in yeast HR dominates. The budding yeast *S. cerevisiae* is the most intensely studied model system for DSB DNA repair. This organism has a classical NHEJ pathway that depends on Ku and DNA ligase IV, as well as Rad50, Mre11 and Xrs2, three proteins that have endo- and exonuclease activities [10],[11].

Agents that cause DSBs often create damaged or non-complementary bases at the break termini [12],[13]. In these circumstances, simple religation cannot occur, and additional factors must be used to process the DNA breaks to create suitable 5′ and 3′ ends for ligation. A nuclease, termed Artemis, has been shown to be important for mammalian NHEJ [14]. Purified Artemis protein possesses single-strand-specific 5′ to 3′ exonuclease activity. In conjunction with the DNA-dependent protein kinase (DNA-PK), Artemis has both 5′ to 3′ and 3′ to 5′ exonuclease activities [14]. *S. cerevisiae* lacks both Artemis and the catalytic component of DNA-PK, and the nuclease(s) and polymerase(s) involved in the yeast NHEJ pathway are not well understood. The Pol X family of DNA polymerases has been implicated in NHEJ, since Pol4, the only Pol X family member in yeast, is required for gap filling in some end configurations [15]. Pol X polymerases appear to be required for NHEJ only when gaps must be filled, indicating that they are not part of the core NHEJ complex [15],[16].

At least six nuclear DNA polymerases have been described in eukaryotic cells that participate in DNA replication and/or repair [17]. Pol1 (designated Pol α or *CDC17* in yeast), Pol2 (Pol ɛ), and Pol3 (Pol δ or *CDC2*) together catalyze the essential functions of DNA replication. Pol2 and Pol3 are also involved in certain DNA repair events, notably nucleotide excision repair [18]. Rev3 (Pol ξ) and Rad30 (Pol η) mediate translesion bypass synthesis in yeast [19]. Pol β is a monomeric polymerase in vertebrates that mediates base excision repair [20],[21]. Moreover, Pol1, Pol2 and Pol3 are required for HR 22–24. Studies in mammals and yeast have provided evidence that Pol α [25], Pol ɛ [26], Pol4 [15] and Pol μ [27] play roles in NHEJ. However, much of these data were obtained through *in vitro* experiments, measuring religation of linear plasmids by cell extracts. Thus, it is not clear which DNA polymerases participate in NHEJ *in vivo* and under what circumstances. For example, DSBs on plasmids with 5′-overhangs do not depend on Pol4 for their repair [16]. Conversely, chromosomal breaks due to HO endonuclease, which generates breaks with 3′ overhangs, do rely on Pol4 for their repair [16].

In this paper we examine the roles of five DNA polymerases (Pol1, Pol2, Pol3, Pol4 and Rev3) in both imprecise end joining and in NHEJ-mediated chromosomal rearrangements in *S. cerevisiae* after a defined HO-induced DSB. These are all members of DNA polymerase families that are conserved from yeast to mammal [17]. Consistent with a previous report [15], we also observed that Pol4 is required for filling in gaps imprecise end joining. On the contrary, Pol2 is involved in deleting bases during imprecise end joining. Both Pol1 and Pol3 are required for chromosomal rearrangements, but Pol3 is not involved imprecise end joining. Our results suggest that most DNA polymerases are involved in NHEJ and each plays a distinct role in the detailed mechanisms of NHEJ.

## Results

### The *In Vivo* NHEJ Assay System

**Figure 1:**
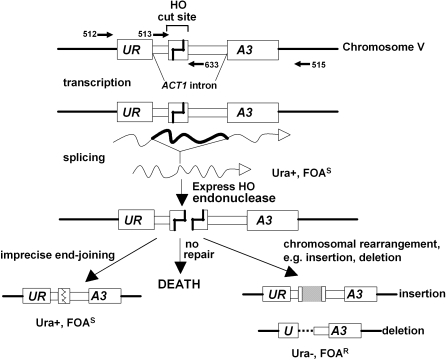
The experimental system. Structure of the *URA3* allele on *S. cerevisiae* chromosome V used for these experiments. The position of various oligonucleotide primers (numbers and half arrows) used for PCR and sequencing are shown. The *ACT1* intron placed into *URA3* is normally spliced, resulting in uracil prototrophy (Ura^+^) and sensitivity to the drug FOA^S^. After creating a DSB within the engineered *ACT1* intron with HO endonuclease, cells either die, are repaired in a way allowing normal splicing, or are repaired in a way that prevents splicing. The latter situation leads to a phenotype of uracil auxotrophy (Ura^−^) and resistance to 5-FOA (FOA^R^).

For our *in vivo* NHEJ assay, a copy of the *ACT1* intron was placed within the *URA3* gene on chromosome V and an HO endonuclease cut site was engineered in the middle of the intron (Figure 1) [28]. A galactose inducible copy of the HO-endonuclease gene is present at the *ADE3* locus on chromosome VII. Other endogenous HO cut sites, as well as the endogenous *ACT1* intron, were deleted. Therefore, upon growth on galactose, the HO endonuclease creates a unique DSB in the middle of *URA3* that must be repaired for cells to survive (Figure 1). Because there are no other *MAT* related sequences in this haploid strain, the DSB cannot be repaired by the HR machinery, and most cells die. Survival is dependent on inefficient NHEJ repair pathways. Precise religation recreates the cut site, which can then be recut by the induced HO endonuclease. The most commonly observed stable repair event is imprecise NHEJ [29]. The imprecision of the end joining eliminates the endonuclease recognition sequence, but these sequence changes do not disrupt splicing because the repair occurs within a nonessential region of the intron. Repair events which interfere with the expression of *URA3*, however, can be identified by selecting for survivors that have become uracil auxotrophs (i.e., resistant to the drug 5-fluoro orotic acid or 5-FOA). Such repair events can include large insertions, deletions extending past the intron sequence, or chromosomal rearrangements such as translocations or inversions that separate the two halves of *URA3* [28],[30],[31]. PCR amplification of the *URA3*::*ACT1* intron::HO cut site allele, Southern blot analysis and/or sequencing of FOA^R^ survivors allow us to distinguish such rearrangements (Figure S1).

### Addition of Bases during Imprecise End Joining Depends On Pol4

To determine which DNA polymerases participate in NHEJ in *S. cerevisiae*, we focused on five different types of DNA polymerases, including two non-essential DNA polymerases (Pol4 and Rev3) and three essential DNA polymerases (Pol1, Pol2 and Pol3). For the nonessential genes, wild-type and deletion mutants were initially grown in rich, galactose-containing, medium (YPGal) to induce HO endonuclease expression. The frequency of imprecise end joining was calculated as the ratio of colonies growing on YPGal compared to colonies growing on rich, glucose-containing medium (YPD) as described in Materials and Methods. The frequency of chromosomal rearrangements (and/or potential *URA3* point-mutations) was estimated as the ratio of colonies growing on FOA-containing media compared to colonies growing on YPD, because insertion or deletion of large fragments results in disruption of splicing and consequent FOA resistance. Additionally, chromosomal translocations and inversions can also disrupt the integrity of the *URA3* gene and cause FOA resistance [31]. It should be noted that the level of analysis carried out in this work (Figure S1) does not definitively distinguish translocations and inversions from insertions.

**Figure 2:**
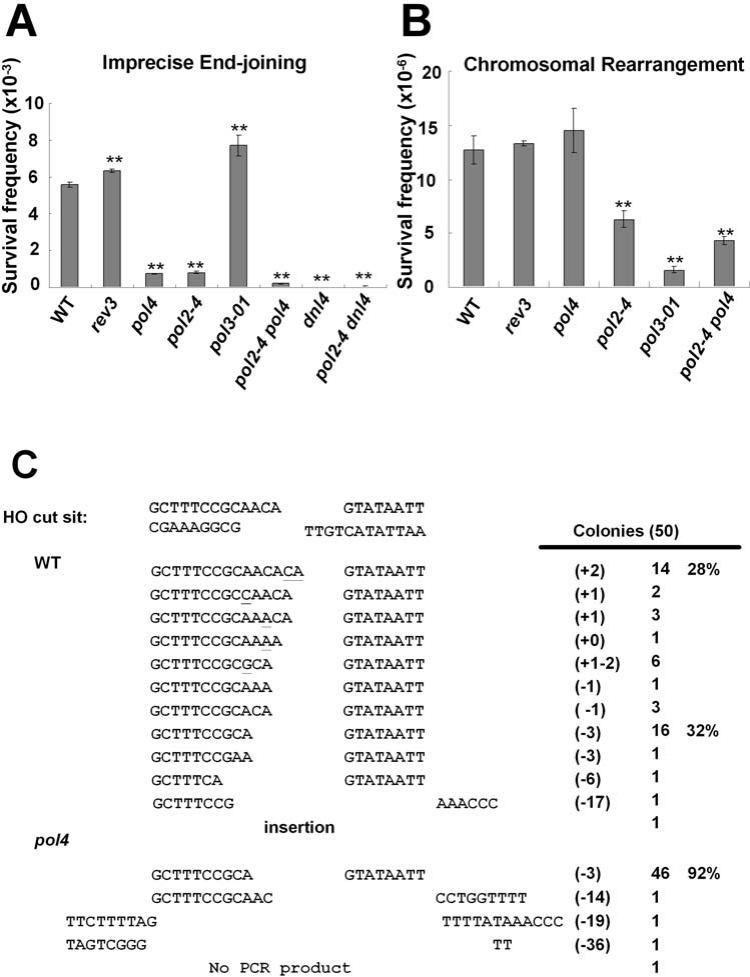
Measurements of NHEJ ability of non-essential DNA polymerases. The efficiency of NHEJ of wild-type and polymerase defective mutants, *rev3*, *pol4* and 3′ to 5′ exonuclease defective mutants, *pol2-4*, *pol3-01*, *pol2-4 pol4*, *dnl4*, and *pol2-4 dnl4* were measured. (A) Frequencies of imprecise end joining. The survival frequency was evaluated by the ratio of YPGal/YPD. (B) Frequency of chromosomal rearrangement was evaluated by the ratio of FOA^R^/YPD. Each experiment was collected from at least four independent clones. *: *P*<0.05; **: *P*<0.01. (C) The sequence analysis of the imprecise end joining events from the WT and *pol4* mutants. The structures of various imprecise end joining repairs were identified at the DSB site. Sequences of the HO cut site are shown, with the resulting 3′ overhanging terminal AACA shown on the cut site. Samples of independent joints were PCR amplified and sequenced. A total of 50 independent survivors on YPGal plates from WT and *pol4* mutant were examined. Bases underlined indicate insertion or mutation. The numbers of inserted (+) and deleted (−) bases were denoted in brackets. N indicates the events of each repair pattern.

We first compared cell survival after a DSB in strains with or without disruption of nonessential polymerases. The wild-type strain, AGY673, had a frequency of imprecise end joining and chromosomal rearrangement events of 5.58×10^−3^ and 1.27×10^−5^ per plated cell, respectively (Figure 2A, 2B, and Table S1). Elimination of Rev3 (*rev3*) did not alter the frequency of imprecise end joining (6.33×10^−3^) or chromosomal rearrangement events (1.33×10^−5^), suggesting that Rev3 is not required for the NHEJ pathway. In contrast, elimination of Pol4 resulted in an 8.6-fold decrease in the frequency of imprecise end joining events, although the frequency of chromosomal rearrangement events was not significantly affected (Figure 2). Therefore, in agreement with previous findings, Pol4 appears to participate in imprecise end joining repair of NHEJ [15].

A previous study showed that the predominant imprecise NHEJ repair products after an HO-induced DSB at the *MAT* ***a*** locus were either addition of two bases (+2 or +CA) or deletion of three bases (−3 or −ACA) [29]. These products are most plausibly caused by a 3′-terminal mismatch (HO (+2)) and by a 3 base flap mismatch (HO (−3)), respectively (see below for detailed description). Thus, we further analyzed the repair in wild-type and *pol4* mutant strains. For wild-type survivors, ∼50% had added bases at the junction (including 28% with the +2 repair pattern) and 46% had deleted bases (including 32% with the −3 pattern) (Figure 2C and Table 1). For *pol4* cells, however, 98% of survivors had deletion of bases at the junction (including 92% with the −3 pattern), while added bases were not observed in 40 independent colonies examined. This pattern confirms that Pol4 is required for addition of bases during imprecise end joining of NHEJ *in vivo* [15]. Interestingly, the absolute frequency of repair involving deletion of bases was also decreased more than 3 fold, from 2.57×10^−3^ in wild-type cells to 7.23×10^−4^ in *pol4* cells (Table S1). These results suggest that Pol4 plays a role in imprecise end joining events *in vivo* where 3′flaps are generated.

**Table 1:** Repair patterns after a DSB at the *URA3*::*ACT1* intron::HO cut site locus of non-essential DNA polymerase mutants.

Strain	Imprecise end joining^a^(%)						Chromosomal rearrangements^b^(%)			
	+CA	−ACA	+base	−base	Others	N^c^	insertions	deletions	mutation	N^c^
WT	28	32	50	46	4	50	28	73	0	40
*rev3*	35	35	55	45	0	20	40	60	0	40
*pol4*	0	92	0	98	2	50	40	60	0	40
*pol2-4*	69	2.2	76	22	2.2	45	45	55	0	40
*pol3-01*	25	40	40	60	0	20	7.5	5	88	40
*pol2-4 pol4*	0	78	0	100	0	50	70	15	15	40
WT (α)^d^	66	6.3	66	34	0	32	nd^d^	nd	nd	nd
*pol2-4* (α)	76	0	87	14	0	37	nd	nd	nd	nd
WT (NZ)^d^	40	7.9	50	47	2.6	38	nd	nd	nd	nd
*pol2-4* (NZ)	74	2.9	85	5.9	8.8	34	nd	nd	nd	nd

a Analysis of survivors grown on YPGal plates.

b Analysis of survivors grown on 5-FOA plates.

c Total events examined.

d nd, not determined; α, α factor arrested; NZ, nocodazole arrested.

### Both Imprecise End Joining and Chromosomal Rearrangement Are Influenced by Essential Polymerases

**Figure 3:**
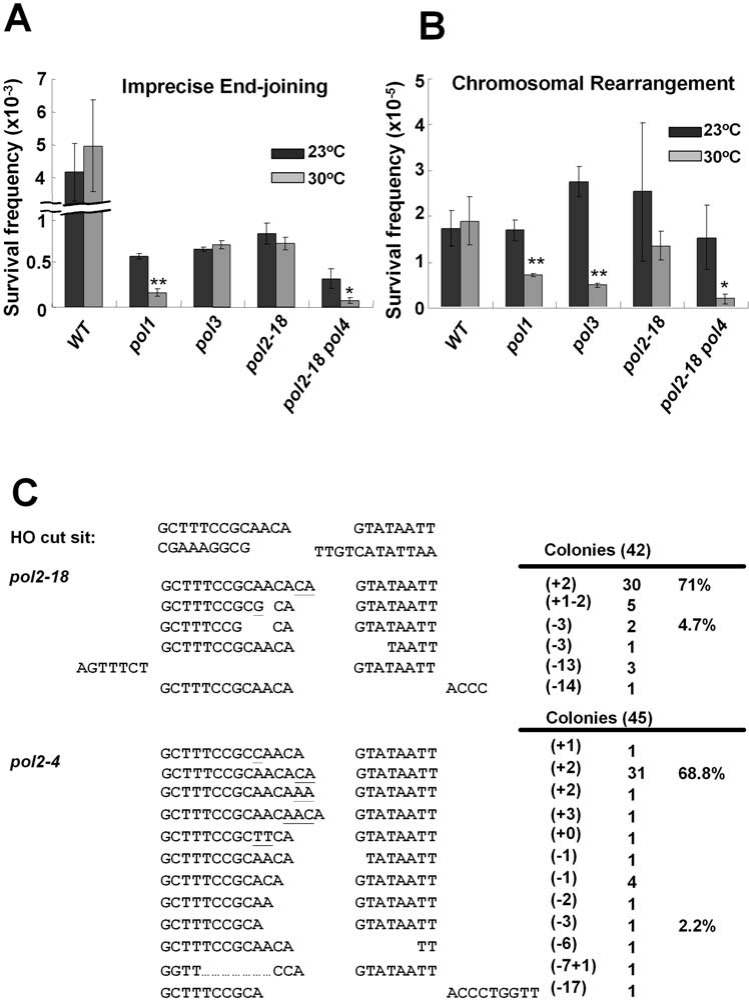
Measurements of NHEJ ability of essential DNA polymerases. The NHEJ assay was performed for *pol1*, *pol3*, *pol2-18* and *pol2-18 pol4* at permissive (23°C) and semi-permissive (30°C) temperatures. (A) Frequencies of imprecise end joining. The survival frequency was evaluated by the ratio of YPGal/YPD. (B) Frequency of chromosomal rearrangement was evaluated by the ratio of FOA^R^/YPD. Each experiment was collected from at least five independent clones. Dark gray bars indicate 23°C incubation and light gray bars indicate 30°C incubation. *: *P*<0.05; **: *P*<0.01. (C) Survivors of *pol2-18* or *pol2-4* mutants from YPGal plates at 30°C were examined. Bases underlined indicate insertion or mutation. The numbers of inserted (+) and deleted (−) bases were denoted in brackets. N indicates the events of each repair pattern.

Three essential DNA polymerases (Pol1, Pol2, and Pol3) are required for DNA replication and HR. To investigate whether they also contribute to NHEJ, temperature-sensitive (ts) mutants with defects in polymerase activity were created in the AGY673 background. As shown in Figure 3A, the presence of any of the ts mutations resulted in a >10-fold drop in survival after a DSB, even at the permissive temperature. Despite this, the frequency of survival and pattern of end joining could be compared for each mutant at the permissive and semi-permissive temperatures, and the patterns of end joining could be compared to the WT strain at the equivalent temperature. As a control we analyzed survival of the ts mutants in the absence of a cut site at *URA3*. We found that strain with no cut sites (STY1553, STY1552 and STY1554) survived equally well at both permissive and semi-permissive temperatures in the absence or presence of HO-endonuclease (Table S2). These results indicate that the change in survival that we observed in the ts mutants after a DSB, was associated with the DSB and its repair, rather than a general effect of the ts mutations, the presence of HO endonuclease, or the growth in galactose versus glucose. Pol1 participates in DNA replication initiation and lagging strand DNA synthesis, as well as HR in yeast [22]–[24],[32]. For Pol1, we found that the frequencies of both imprecise end joining (YPGal/YPD) and chromosomal rearrangements (FOA/YPD) were reduced more than 50% compared with those cultured at permissive temperature (23°C), a decrease that was significant to a *P*<0.01 (Figure 3A, 3B, and Table S2). However, there was no significant difference in the pattern of imprecise end joining events or in the percentages of insertions or deletions (Table 2). These results suggest that the effect of Pol1 on NHEJ is indirect.

**Table 2:** Repair patterns after a DSB at the *URA3*::*ACT1* intron::HO cut site locus of essential DNA polymerase mutants.

Strain	Tm(°C)	Imprecise end joining^a^(%)						Chromosomal rearrangements^b^(%)			
		+CA	−ACA	+base	−base	Others	N^c^	insertions	deletions	mutation	N^c^
WT	23	15	25	35	65	0	20	75	25	0	20
	30	40	25	40	50	10	20	50	50	0	20
*pol1*	23	35	35	50	50	0	20	55	45	0	40
	30	40	35	60	40	0	20	60	40	0	40
*pol3*	23	25	40	45	55	0	20	80	20	0	40
	30	30	35	40	60	0	20	80	20	0	40
*pol2-18*	23	42	28	64	36	0	50	75	7	18	40
	30	71	4.7	83	17	0	42	88	7	5	40
*pol2-18*	23	0	80	0	100	0	50	60	7	33	40
*pol4*	30	0	74	0	100	0	50	53	17	30	40

a Analysis of survivors grown on YPGal plates.

b Analysis of survivors grown on 5-FOA plates.

c Total events examined.

Pol3 is essential for both leading strand and lagging strand DNA synthesis [32]. Interestingly, we found that while the frequency of imprecise end joining did not change, the frequency of chromosomal rearrangements was significantly reduced in the *pol3* mutant at the semi-permissive temperature (Figure 3A, 3B, and Table S2). The repair pattern of the *pol3* ts mutant showed no obvious difference compared to WT for either end joining or chromosomal rearrangements (Table 3), implying that the major consequence of the *pol3* ts mutation at the semi-permissive temperature was a defect in the ability to generate complex chromosomal rearrangements.

**Table 3:** Yeast strains used in this study.

Strain	Genotype	Source
AGY628	*ho,hml::ADE1,mata::*hisG, *hmr::ADE, ade1, lys5, trp1::*hisG, *ade3::GAL-HO, ura3-52, LEU2*, intronless *ACT1*	[44]
AGY673	AGY628 *URA3*::ai::HO cut site	[44]
YHA322	*MATα pol2-3::LEU2 leu2-3,112 ura3-52 trp1-289 his4* [YCp*pol2-18*]	[46]
STY891	AGY673 *pol4::TRP1*	This study
STY890	AGY673 *rev3::TRP1*	This study
STY969	AGY673 *pol1* (*cdc17-1*)	This study
STY970	AGY673 *pol3* (*cdc2-2*)	This study
STY966^a^	AGY673 *pol2::LEU2* [YCp*pol2-18*]	This study
STY1358	AGY673 *pol2-4*	This study
STY1359	AGY673 *pol3-01*	This study
STY1356^b^	AGY673 *pol2::LEU2* [YCp*pol2-18*] *pol4*::*TRP1*	This study
STY1360^c^	AGY673 *pol2-4 pol4*::*TRP1*	This study
STY1544	AGY673 *dnl4::*KanMX4	This study
STY1546	AGY673 *pol2-4 dnl4::*KanMX4	This study
STY1549	AGY673 *pol3* (*cdc2-2*) *ura3*Δ	This study
STY1550	AGY673 *pol1* (*cdc17-1*) *ura3*Δ	This study
STY1551	AGY673 *pol2::LEU2* [YCp*pol2-18*] *ura3Δ*	This study
STY1552	AGY673 *pol3* (*cdc2-2*) *URA3::*ai	This study
STY1553	AGY673 *pol1* (*cdc17-1*) *URA3::*ai	This study
STY1554	AGY673 *pol2*::*LEU2* [YCp*pol2-18*] *URA3::*ai	This study

a From three backcrosses of YHA322 with AGY673.

b *pol4* of STY966.

c *pol4* of STY1358.

### Pol2 Contributes to Imprecise End Joining through Its 3′ to 5′ Exonuclease Activity

Pol2 interacts with PCNA in S phase and is required for the S-phase checkpoint, for assembly of replication complexes at origins, and for leading strand replication [33],[34]. A *pol2* polymerase mutant (*pol2-18*) at the semi-permissive temperature showed no obvious difference in the frequency of imprecise end joining or chromosomal rearrangements compared with the same strain at 23°C (Figure 3A and 3B). Sequence analysis of the imprecise end joining repair pattern, however, revealed that the proportion of −3 events was reduced more than 5 fold at 30°C compared to 23°C, while the proportion of +2 events was nearly doubled (Table 3). These data suggest that Pol2 is particularly involved in generating the −3 product during imprecise end joining repair.

A likely step in the creation of the −3 end joining product is deletion of the 3 base 3′ flaps from both broken ends. Pol2 might directly participle in this step through its associated exonuclease activity. To examine this possibility, we constructed *pol2-4* and *pol3-01* mutant strains which are defective in the 3′ to 5′ exonuclease activity but do not affect the DNA polymerase activity of Pol2 and Pol3, respectively [35],[36]. These are both non-lethal mutant strains and both have been shown to display mutator phenotypes [35],[37]. Interestingly, *pol2-4* but not *pol3-01* affects the frequency of imprecise end joining (Figure 2A), and a reduction of the −3 pathway was observed in the *pol2-4* strain, but not in the *pol3-01* strain (Figure 3C and Table 1). The absolute frequency of base loss during repair was decreased more than 10 fold, from 2.57×10^−3^ in wild-type cells to 1.83×10^−4^ in *pol2-4* cells (Table S1). In particular the absolute frequency of the −3 product was decreased over 98-fold, from 1.79×10^−3^ in wild-type cells to 1.82×10^−5^ in *pol2-4* cells (Table S1). These results support our hypothesis that the 3′ to 5′ exonuclease activity of Pol2 is involved in the processing step that degrades the 3′ flaps to create the −3 end joining product. Further, we confirmed that the events we observed in the *pol2-4* strain were in fact due to NHEJ. We compared survival in isogenic *dnl4* and *pol2-4 dnl4* yeast cells relative to the wild type and *pol2-4* strains (Figure 2 and Table S1), and found an ∼100 fold drop in the absence of *dnl4*, a key component of the NHEJ pathway. Of note, we also observed that 87.5% of FOA resistant survivors in *pol3-01* cells showed the parental size product at the DSB site (Table 1). Sequencing results of the HO cut site junctions in twenty independent survivors revealed that the junctions of each survivor contain different imprecise end joining sequences, suggesting that they are not siblings from a single event (data not shown). Since these changes should not affect splicing of the *ACT1* intron, the observed FOA resistance likely resulted from mutations in the *URA3* coding region. These data support the previously described mutator phenotype of *pol3-01* [35],[36].

Since both Pol2 and Pol4 affect the frequency and pattern of imprecise end joining after an HO-induced DSB, we next asked whether their effects were additive. To this end we tested both *pol2-18 pol4* and *pol2-4 pol4* double mutant strains with our assay (Figures 2 and 3, and Tables 2 and 3). Strikingly, both *pol2-18 pol4* and *pol2-4 pol4* double mutant strains showed further reduction of imprecise end joining events compared to those of the single mutations (Figures 2A and 3A). Examining the pattern of repair, we found that the −3 product predominated in the double mutants (Tables 2, 3, S1 and S2). These results suggest that the +2 product pathway is completely Pol4-dependent. The absolute frequency of repair with deletions decreased from 7.23×10^−4^ in *pol4* cells to 2.58×10^−4^ in *pol2-4 pol4* cells (Table S1). Therefore, in contrast to Pol4, Pol2 is not the exclusive nuclease for processing the −3 product pathway.

Our data also provide evidence for interplay between Pol4 and Pol2 in base deletion and addition during imprecise end joining repair. Although Pol4 has its major effect on base addition, the absolute frequency of base deletion products decreased 3.6 fold, from 2.57×10^−3^ in wild-type cells to 7.23×10^−4^ in *pol4* cells. Conversely, whereas Pol2 is associated with base deletions, the absolute frequency of base addition products decreased >44 fold, from 2.79×10^−3^ in wild-type cells to 6.24×10^−5^ in *pol2-4* cells (Table S1).

### Lack of Cell Cycle Dependence of Pol2-Mediated 3′-End Processing of NHEJ

Moore and Haber [29] have shown that specific NHEJ repair products have a cell cycle dependence, with the proportion of −3 products increasing and the proportion of +2 products decreasing in G1. One possible explanation for our observed Pol2-mediated 3′-end processing defect is that the cell cycle in the *pol2* mutants is altered, possibly even arrested at some stage. In that case, the decreased proportion of −3 products could be related to the cell cycle stage, rather than a direct consequence of Pol2's role in NHEJ. To test whether Pol2-mediated 3′-end processing of NHEJ varies by cell cycle stage, we induced HO cleavage after arresting wild-type or *pol2* cells either at G1 or G2/M. As shown in Tables 2 and S1, *pol2-4* cells exhibited a reduced frequency of repair via base deletion, regardless of whether the cells were non-synchronized, arrested in G1 or arrested in G2/M. These results indicate that reduction of base deletion during NHEJ in *pol2* cells is not cell cycle dependent.

## Discussion

From yeast to humans, NHEJ plays a role in the repair of DSBs. The range of proteins required for the various forms of NHEJ, however, have not yet been defined. DNA polymerases should be important contributors to DNA repair. Gaps often occur which require reconstruction and the nuclease activities associated with DNA polymerases can be utilized for processing repair intermediates. The essential DNA polymerases Pol1, Pol2 and Pol3 have previously been implicated as being required for HR-type DSB repair [22]–[24]. Pol4 was the first DNA polymerase found to participate in repairing 3′ overhangs during NHEJ in yeast [15]. Interestingly, two Pol4 homologs, Pol μ and Pol λ, have also been implicated in NHEJ in humans [27],[38],[39]. The fact that Pol4 directly interacts with DNA ligase IV implies that Pol4 might recruit other NHEJ factors to the DSB sites [11],[40],[41]. Indeed, we have observed, by chromatin immunoprecipitation, that Pol4 is present at DSB sites (Tseng and Teng, unpublished observation).

In this study, we aimed to clarify the in *vivo* roles of DNA polymerases in NHEJ-type DSB repair. Of the DNA polymerases we examined, only Rev3, showed no involvement in some aspect of NHEJ. This exception is interesting in that Rev3 has been shown, by chromatin immunoprecipitation, to localize to the site of HO-induced DSBs [42]. Pol4 is specifically required for adding bases during imprecise end joining; Pol3 is required for some unknown aspect of generating complex chromosomal rearrangements; both Pol1 and Pol2 play roles in both imprecise end joining and chromosomal rearrangements. Although Pol4has a primary rolein resynthesizing gaps and Pol2contributes to deleting the flaps at imprecise pairing sites at a DSB, we found that Pol4and Pol2can also influence, respectively, base deletion and addition during imprecise end joining repair. These results provide evidence for interplay between Pol4 and Pol2. We speculate that eliminating either one of these polymerases may influence the ability of the other to repair the DSB. This may also account for the observed overall reduction in survival in the presence of any of the ts mutations, even at the permissive temperature (Figure 3A). Even subtle changes in these essential proteins could tip the balance away from successful repair. Our study reflects the diversity, collaboration, and redundancy of multiple DNA polymerases, both essential and non-essential, in eukaryotic repair processes. DSBs generate different DNA end structures, which need to be recognized and repaired by complexes that likely include more than one DNA polymerase.

**Figure 4:**
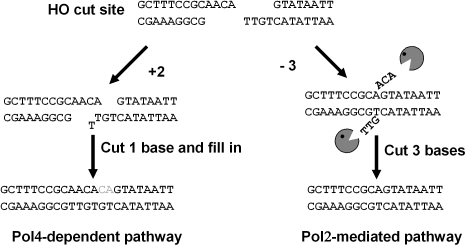
Proposed pathways for imprecise end joining of the HO endonuclease induced DSB at *MATa*. The 4-bp 3′ overhanging sequence resulting from HO endonuclease cleavage is indicated. The two ends must be brought together into synapsis. Precise religation would lead to re-cutting. Possible end processing and subsequent alignment of complementary base pairs that would lead to insertions or deletions are shown. When 3′ overhangs mispair, Pol4 is required to fill in the gap [15],[16]. When 3′ flaps are generated by mis-alignment, the 3′ to 5′ exonuclease activity of Pol2 is the predominate exonuclease to remove the flaps.

Our data demonstrates that Pol2, and more specifically, the 3′ to 5′ exonuclease activity of Pol2, plays a significant role in generating imprecise NHEJ joints that require removal of 3′ terminal flaps (Figure 4). However, unlike filling in gaps resulting from 3′ terminal mismatches, which seems to be an exclusive function of Pol4, flap removal can be carried out by nucleases other than Pol2, although at reduced efficiency. The frequencies of imprecise end joining in *pol2-18 pol4* and *pol2-4 pol4* double mutant strains were significantly decreased compared to those of wild-type or either single mutant, but in both double mutant strains DSB repair was predominantly through the −3 pathway of imprecise end joining. Since the *pol4* mutant used in this study contains a complete truncation of the *POL4* open reading frame, our data imply that other unidentified 3′ to 5′ exonucleases might be utilized in this NHEJ process. Further studies with additional mutant genes should help to delineate the roles and compartmentalization of particular repair factors in different aspects of NHEJ.

## Materials and Methods

### Yeast Strain and Plasmid Constructions

General yeast manipulations were performed as described [43]. The *S. cerevisiae* strains used in this study are listed in Table 3. Yeast strains used in the study were derivatives of AGY628 or AGY673 [44]. The fragments of *POL4* (coding sequence 286∼969) and *REV3* (coding sequence 2652∼3863) were PCR-amplified from yeast genomic DNA and cloned into *Pvu*II digested pRS304. pRS304*pol4* and pRS304*rev3* were linearized by *Afl*II and *Hpa*I, respectively. And these linearized YIPs were transformed into AGY673 using single crossover approach [43] to create *pol4* (STY891) and *rev3* (STY890) mutant strains. pSD218 [45] (kindly provided by Dr. Daniel E Gottschling) was used as previously described to create the *pol1*(*cdc17-1*) mutant in AGY673 (STY969). pST738 was constructed by ligating PCR-amplified *pol3*(*cdc2-2*) (coding sequence −600∼1950) from yeast strain UCC5898 (generously provided by Dr. Daniel E Gottschling) [45] to pRS306. *Bgl*II linearized pST738 was used to transform AGY628. *pol3* of AGY628 was obtained by the two step pop in and pop out method, selecting first for Ura^+^ and then for Ura^−^ by FOA and screened for temperature-sensitivity at 37°C for *pol3* of AGY628. *pol3* of AGY628 was then transformed with the PCR fragment of *URA3::ai::HO* to create the *pol3* ts mutant of AGY673 (STY970). The *pol2-18* mutant strain (STY966) was constructed by backcrossing YAH322 [46] (kindly provided by Dr. Akio Sugino) with AGY673 three times, each time selecting for spores with temperature sensitivity. YIpBI and YIpAM26 (kindly provided by Dr. Akio Sugino) were used to create *pol2-4* (STY1358) and *pol3-01* (STY1359) mutants respectively, as previously described by selecting Ura+ transformants using the single crossover replacement method, and then FOA selection for popouts removed the wild-type sequence [37],[43]. Genotype was confirmed by PCR and sequencing. STY1356 (*pol2-18 pol4*) and STY1360 (*pol2-4 pol4*) were constructed by disrupting *POL4* in STY966 and STY1356 using pRS304*pol4*, respectively. A *ura3* deletion fragment was PCR amplified from an FOA-resistant clone of AGY673. “No cut” site controls were created using the double crossover approach [43] by transforming the *ura3* deletion fragment into STY969, STY973 and STY966 and selecting for intergrants on FOA plates to obtain STY1553, STY1552 and STY1554, respectively. The *dnl4* strain was constructed by transformation of a PCR product into AGY673 and STY1358 using the genomic DNA from a BY4741 *dnl4* strain (Invitrogen) as a template and oligonucleotides flanking the *DNL4* gene as primers. Transformants with double crossover at *DNL4* were selected for G418 resistance and insertion sites were confirmed by PCR. All primer sequences for PCR are available upon request.

### Media and Growth Conditions

Yeast cells were grown in yeast extract-peptone-dextrose (YPD) or synthetic complete media (SC) with appropriate amino acids missing [47]. Yeast extract-peptone-galactose (YEP-galactose) and yeast extract-peptone-raffinose (YEP-raffinose) contain 2% galactose (w/v) and 2% raffinose (w/v), respectively, instead of dextrose (2%). 5- fluoro-orotic acid (5-FOA) plates are SC glucose plates supplemented with 1 mg/ml of 5-FOA [48],[49].

### The NHEJ Assay

The NHEJ assay system was established in the Gabriel lab [28]. This system contains a positive selection for chromosomal changes, including insertions, deletions, translocations and inversions, associated with repair of a defined chromosomal DSB. As shown in Figure 1, a functional *URA3* allele was created on chromosome V, which contains a copy of the *ACT1* intron as well as the Y–Z junction from the *MAT*a locus. The Y-Z junction includes the recognition sequence and cleavage site for the HO endonuclease, was created on chromosome V. All *MAT* related sequences had been deleted and this strain contains an integrated galactose-inducible HO endonuclease gene.

### Induction of the HO Endonuclease, Measurement of DSB Repair Efficiency (Survival Frequency), and 5-FOA Resistance Frequency

For non-essential polymerase mutants, multiple independent colonies from each strain were grown at 30°C in YEP-raffinose liquid medium to a final concentration of OD_595_ ∼1. Appropriate dilutions of cells were then plated on YPD or YEP-galactose (YPGal) plates. Colonies were counted after 4 days of growth. Colonies on the YEP-galactose plates were replica plated onto synthetic complete 5-FOA-containing media to measure the frequency of 5-FOA resistance among survivors of HO endonuclease induction. For essential polymerase mutants, multiple independent colonies from each strain were grown at 23°C in YEP-raffinose liquid medium to a final concentration of OD_595_ ∼1. Appropriate dilutions of cells were then plated on YPD or YPGal plates and incubated at 23°C or 30°C. Colonies were counted after four days of growth. Alternatively, yeast cells were diluted to YPGal liquid medium to OD_595_ ∼0.5, incubated at 23°C or 30°C for 20 hours, and then serial diluted to plate on YPGal and FOA plates. The frequency for imprecise end joining is the ratio of the number of colonies growing on YPGal *vs.* YPD from per ml of culture. The frequency of chromosomal rearrangements is the number of colonies growing on 5-FOA *vs.* YPD from per ml of culture. It was notable that colony sizes were smaller and much more heterogeneous on YPGal and FOA plates from all ts mutants, at both the permissive and semi-restrictive temperatures, necessitating an arbitrary cutoff for tiny colonies that were hard to score.

All values are expressed as means (±) standard error. Differences between groups were tested using the student's t-test. For non-essential polymerase mutants, significance tests were compared against frequency of wild type. For essential polymerase mutants, significance tests were compared against frequencies at permissive temperature. Samples of survivors growing on YPGal were analyzed by PCR using primer RAG512 and RAG515 flanking the *URA3* gene (Figure 1) [28], and PCR products were sequenced using internal primer RAG513 or RAG633 (Figure 1) [28]. Survivors growing on FOA were further analyzed by Southern blot analysis (Figure S1). For preparing probes, the *URA3*::*ACT1* intron::HO cut site fragment was amplified by PCR using primer RAG512 and RAG515. The 799 (probe A) and 878 (probe B) base pairs *Xho*I-digested PCR fragments were used as probes for Southern hybridization. In most cases, 40 survivors from four to seven independent cultures were examined for each strain. Based on Southern blot analysis, a rearrangement was termed an insertion if we observed two or more hybridizing bands, a deletion if we observed only a single band, and a mutation if the single band was the same size as the parent strain. Note that potential translocations or inversions were read as insertions. Further, very small insertions could be mis-read as mutations because of minimal change in band size, although we did not see evidence of this after sequencing twenty same size products from the *pol3-01* strain (data not shown).

### Cell-Cycle Experiments

Multiple independent colonies from each strain were grown at 30°C overnight in YEP-raffinose liquid medium. Yeast cells were diluted to YEP-raffinose liquid medium to OD_595_ 0.1 to refresh for three hours, incubated at 30°C for additional four hours in 100 µM α-factor or 20 µg/ml nocodazole. Cultures were split and half continued to grow in YEP-raffinose with α-factor or nocodazole while the other half received galactose to a final concentration of 2% for one hour. Yeast cells from YEP-raffinose and YPGal were then serial diluted on YPD and YPGal plates, respectively. The frequency of imprecise end joining was determined as the ratio of the number of colonies growing on YPGal *vs.* YPD from per ml of culture.

## Supporting Information

Figure S1 Southern blot analysis of 5-FOA-resistant survivors after HO endonuclease induced.
(0.55 MB DOC)

Table S1 Quantitative analysis of repair events at the *URA3::ACT1* intron::HO cut site locus of non-essential DNA polymerase mutants.
(0.05 MB DOC)

Table S2 Quantitative analysis of repair events at the *URA3::ACT1* intron::HO cut site locus of essential DNA polymerase mutants.
(0.06 MB DOC)
