# Supplementary material for: Proofreading Activity of DNA Polymerase Pol2 Mediates 3′-End Processing during Nonhomologous End Joining in Yeast
Source: PLoS Genet. 2008 Apr 25;4(4):e1000060. doi: 10.1371/journal.pgen.1000060 (PMC2312331; doi:10.1371/journal.pgen.1000060)
Supplement: Figure S1 — Southern blot analysis of 5-FOA-resistant survivors after HO endonuclease induced. (0.55 MB DOC) [file pgen.1000060.s001.doc]

**
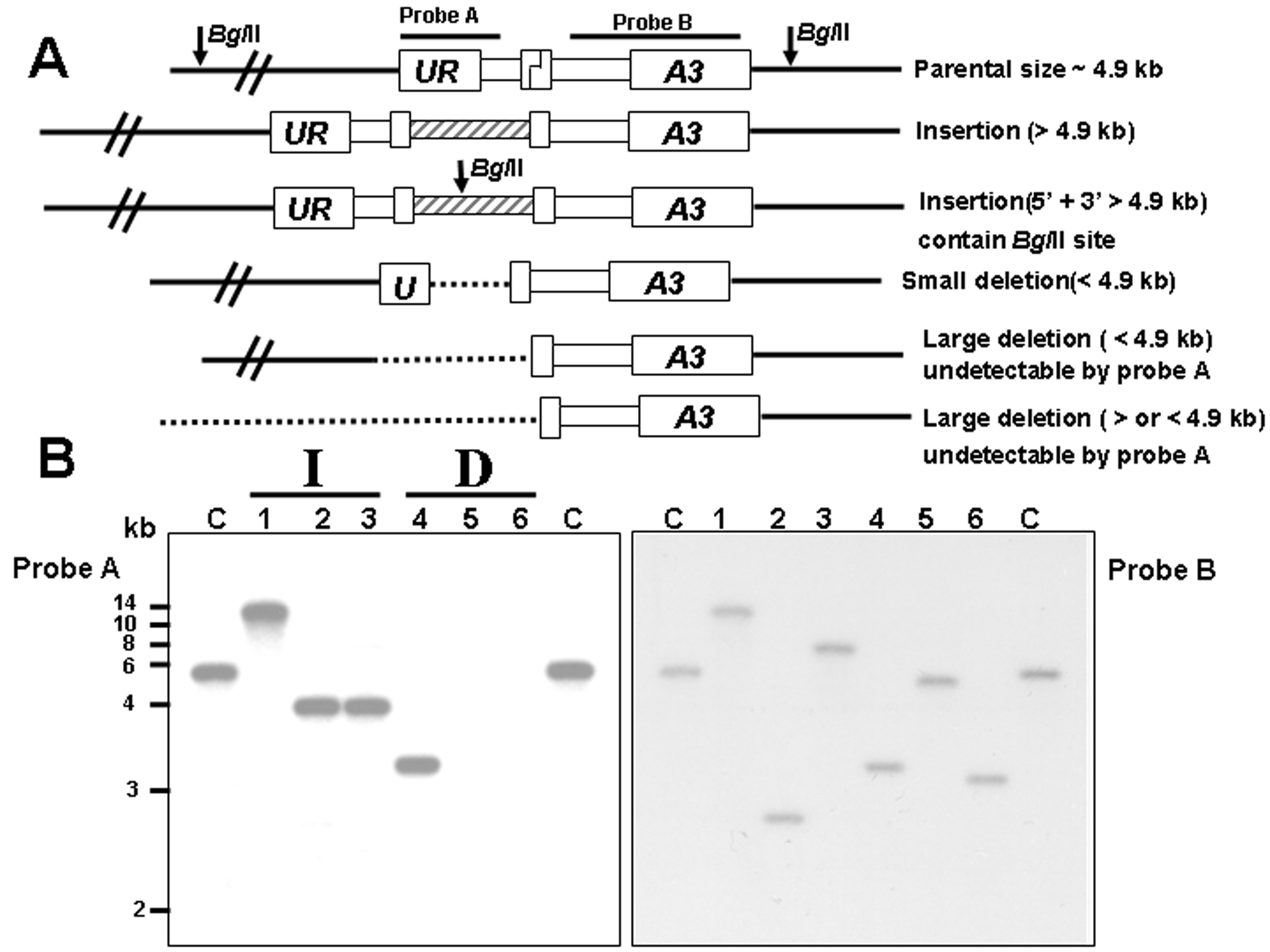
**

**Fig. S1. Southern blot analysis of 5-FOA-resistant survivors after HO endonuclease induced.** **(A)** Scheme of large fragment rearrangement identified in the NHEJ assay system. Various patterns of insertion and deletion events were recovered from FOA plates. Probe A and B were used for Southern blot analysis. **(B)** Genomic DNA was digested with *Bgl*II and then separated by a 1% agarose gel. Left panel shows a membrane hybridized with the probe A; right panel indicates the same membrane hybridized with the probe B. (1) Insertion without any *Bgl*II site, (2) and (3) insertion with a *Bgl*II site, (4) small fragment deletion, (5) and (6) large fragment deletion. Control (C): AGY673 (WT). I: insertion events; D: deletion events.
