# Supplementary material for: Proofreading Activity of DNA Polymerase Pol2 Mediates 3′-End Processing during Nonhomologous End Joining in Yeast
Source: PLoS Genet. 2008 Apr 25;4(4):e1000060. doi: 10.1371/journal.pgen.1000060 (PMC2312331; doi:10.1371/journal.pgen.1000060)
Supplement: Table S1 — Quantitative analysis of repair events at the URA3::ACT1 intron::HO cut site locus of non-essential DNA polymerase mutants. (0.05 MB DOC) [file pgen.1000060.s002.doc]

**Table S1. Quantitative analysis of repair events at the *URA3*::*ACT1* intron::HO cut site locus of non-essential DNA polymerase mutant**s

| Strain | Imprecise end joining | | | | | | Chromosomal rearrangements | |
| --- | --- | --- | --- | --- | --- | --- | --- | --- |
|  | Survival Frequency^a^ | *P* value | +CA^c^ | -ACA^c^ | + base^c^ | -base^c^ | Survival Frequency^b^ | *P* value |
| WT | 5.58 x 10^-3^ (0.12) |  | 1.56 x 10^-3^ | 1.79 x 10^-3^ | 2.79 x 10^-3^ | 2.57 x 10^-3^ | 1.27 x 10^-5^ (0.11) |  |
| *rev3* | 6.33 x 10^-3^ (0.1) | <0.01 | 2.22 x 10^-3^ | 2.22 x 10^-3^ | 3.48 x 10^-3^ | 2.84 x 10^-3^ | 1.33 x 10^-5^ (0.02) | 0.47 |
| *pol4* | 7.38 x 10^-4^ (0.43) | <0.01 | 0 | 6.79 x 10^-4^ | 0 | 7.23 x 10^-4^ | 1.45 x 10^-5^ (0.2) | 0.07 |
| *pol2-4* | 8.25 x 10^-4^ (0.64) | <0.01 | 5.68 x 10^-4^ | 1.82 x 10^-5^ | 6.24 x 10^-4^ | 1.83 x 10^-4^ | 6.25 x 10^-5^ (0.08) | <0.01 |
| *pol3-01* | 7.71 x 10^--3^ (0.56) | <0.01 | 1.93 x 10^--3^ | 3.08 x 10^--3^ | 4.63 x 10^--3^ | 3.08 x 10^--3^ | 1.58 x 10^-6^ (0.29) | <0.01 |
| *pol2-4* *pol4* | 2.58 x 10^-4^ (0.47) | <0.01 | 0 | 2.01 x 10^-4^ | 0 | 2.58 x 10^-4^ | 4.28 x 10^-6^ (0.37) | <0.01 |
| *dnl4* | 6.31 x 10^-6^ (1.74) | <0.01 | nd^d^ | nd | nd | nd | nd | nd |
| *pol2-4 dnl4* | 1.20 x 10^-5^ (0.69) | <0.01 | nd | nd | nd | nd | nd | nd |
| WT(NS)^d^ | 5.97 x 10^-3^ (0.4) |  | nd | nd | nd | nd | nd | nd |
| *pol2-4*(NS) | 2.17 x 10^-3^ (0.5) | <0.01 | nd | nd | nd | nd | nd | nd |
| WT (α)^d^ | 3.43 x 10^-3^ (0.75) |  | 2.26 x 10^-3^ | 2.16 x 10^-4^ | 2.26 x 10^-3^ | 1.17 x 10^-3^ | nd | nd |
| *pol2-4* (α) | 2.23 x 10^-3^ (0.15) | 0.08 | 1.69 x 10^-3^ | 0 | 1.92 x 10^-3^ | 3.12 x 10^-4^ | nd | nd |
| WT (NZ)^d^ | 5.37 x 10^-3^ (0.75) |  | 2.15 x 10^-3^ | 4.24 x 10^-4^ | 2.69 x 10^-3^ | 2.52 x 10^-3^ | nd | nd |
| *pol2-4* (NZ) | 2.17 x 10^-3^ (0.12) | <0.01 | 1.61 x 10^-3^ | 6.29 x 10^-5^ | 1.84 x 10^-3^ | 1.28 x 10^-4^ | nd | nd |

^a^ The mean survival frequency was determined by the ratio of colonies appearing on YPGal plates to those growing on YPD plates, based on 4 independent trials. Standard deviation in parentheses is expressed in the same units.

^b^ The mean survival frequency was determined by the ratio of colonies appearing on 5-FOA plates to those growing on YPD plates.

^c^ The absolute frequencies of different joints (survival frequency x joint frequency among survivors)

^d^ nd, not determined; NS, nonsynchronized; α, α factor arrested; NZ, nocodazole arrested.
