# Supplementary material for: Proofreading Activity of DNA Polymerase Pol2 Mediates 3′-End Processing during Nonhomologous End Joining in Yeast
Source: PLoS Genet. 2008 Apr 25;4(4):e1000060. doi: 10.1371/journal.pgen.1000060 (PMC2312331; doi:10.1371/journal.pgen.1000060)
Supplement: Table S2 — Quantitative analysis of repair events at the URA3::ACT1 intron::HO cut site locus of essential DNA polymerase mutants. (0.06 MB DOC) [file pgen.1000060.s003.doc]

**Table S2. Quantitative analysis of repair events at the *URA3*::*ACT1* intron::HO cut site locus of essential DNA polymerase mutant**s

| Strain | Tm (^o^C) | Imprecise end joining | | | | | | Chromosomal rearrangements | |
| --- | --- | --- | --- | --- | --- | --- | --- | --- | --- |
|  |  | Survival Frequency^a^ | *P* value | +CA^c^ | -ACA^c^ | + base^c^ | -base^c^ | Survival Frequency^b^ | *P* value |
| WT | 23 | 4.16 x 10^-3^(0.09) |  | 6.24 x 10^-4^ | 1.04 x 10^-3^ | 1.46 x 10^-3^ | 2.7 x 10^-3^ | 1.74 x 10^-5^(0.38) |  |
|  | 30 | 4.96 x 10^-3^(1.40) | 0.07 | 1.98 x 10^-3^ | 1.24 x 10^-3^ | 1.98 x 10^-3^ | 2.48 x 10^-3^ | 1.90 x 10^-5^(0.51) | 0.12 |
| *Pol1* | 23 | 5.41 x 10^-4^(0.37) |  | 1.80 x 10^-4^ | 1.80 x 10^-4^ | 2.71 x 10^-4^ | 2.71 x 10^-4^ | 1.70 x 10^-5^(0.22) |  |
|  | 30 | 1.82 x 10^-4^(0.50) | <0.01 | 7.28 x 10^-5^ | 6.37 x 10^-5^ | 1.10 x 10^-4^ | 7.28 x 10^-5^ | 7.14 x 10^-6^(0.33) | <0.01 |
| *pol3* | 23 | 5.99 x 10^-4^(0.24) |  | 1.50 x 10^-4^ | 2.40 x 10^-4^ | 2.70 x 10^-4^ | 3.29 x 10^-4^ | 2.74 x 10^-5^(0.33) |  |
|  | 30 | 6.87 x 10^-4^(0.63) | 0.07 | 2.06 x 10^-4^ | 2.40 x 10^-4^ | 2.75 x 10^-4^ | 4.12 x 10^-4^ | 5.01 x 10^-6^(0.39) | <0.01 |
| *pol2-18* | 23 | 8.95 x 10^-4^(1.71) |  | 3.76 x 10^-4^ | 2.24 x 10^-4^ | 5.73 x 10^-4^ | 3.22 x 10^-4^ | 2.53 x 10^-5^(1.5) |  |
|  | 30 | 7.04 x 10^-4^(1.35) | 0.07 | 5.00 x 10^-4^ | 3.31 x 10^-5^ | 6.22 x 10^-4^ | 1.18 x 10^-4^ | 1.36 x 10^-5^(1.35) | 0.09 |
| *pol2-18* *pol4* | 23 | 2.91 x 10^-4^(1.6) |  | 0 | 2.33 x 10^-4^ | 0 | 2.91 x 10^-4^ | 1.54 x 10^-5^(0.7) |  |
|  | 30 | 6.35 x 10^-5^(2.8) | <0.05 | 0 | 4.70 x 10^-5^ | 0 | 6.35 x 10^-5^ | 2.06 x 10^-6^(1.0) | <0.05 |
| *pol1 URA3::*ai*^d^* | 23 | 0.86 |  | nd^e^ | nd | nd | nd | nd | nd |
|  | 30 | 0.88 | 0.46 | nd | nd | nd | nd | nd | nd |
| *pol3 URA3::*ai*^d^* | 23 | 0.95 |  | nd | nd | nd | nd | nd | nd |
|  | 30 | 0.95 | 0.8 | nd | nd | nd | nd | nd | nd |
| *pol2-18 URA3::*ai*^d^* | 23 | 0.89 |  | nd | nd | nd | nd | nd | nd |
|  | 30 | 0.90 | 0.94 | nd | nd | nd | nd | nd | nd |

^a^ The mean survival frequency was determined by the ratio of colonies appearing on YPGal plates to those growing on YPD plates, based on at least 5 independent trials. Standard deviation in parentheses is expressed in the same units.

^b^ The mean survival frequency was determined by the ratio of colonies appearing on 5-FOA plates to those growing on YPD plates.

^c^ The absolute frequencies of different joints (survival frequency x joint frequency among survivors)

^d^ No HO cut sit (*URA3::*ai)

^e^ nd, not determined.
